# Late Repair of Native Pulmonary Valve for Severe Pulmonary Regurgitation After Transannular Patch Repair in Tetralogy of Fallot

**DOI:** 10.1016/j.atssr.2025.04.023

**Published:** 2025-05-17

**Authors:** John L. Wiegand, Zainab B. Ezzi, Maher N. Abadeer, James A. Quintessenza, Brandi B. Scully

**Affiliations:** 1 Department of Pediatrics, University of South Florida, Tampa, Florida; 2 Division of Pediatric Cardiology, Johns Hopkins All Children’s Hospital, St Petersburg, Florida; 3 Division of Pediatric Cardiac Surgery, Johns Hopkins All Children’s Hospital, St Petersburg, Florida

## Abstract

Despite advances in surgical repair of tetralogy of Fallot, long-term complications such as pulmonary regurgitation occur frequently. We present a case of a 10-year-old boy who underwent transannular repair of tetralogy of Fallot at 6 months of age. Chronic severe pulmonary regurgitation necessitated late primary repair of his pulmonary valve with resection of the transannular patch, improving his right ventricular function. Postoperative recovery was uneventful. This case underscores the importance of high-resolution preoperative imaging in guiding surgical management and timely intervention for complications after repair and highlights an alternative strategy to minimize the need for repeated valve replacements.

Tetralogy of Fallot (TOF), the most common cyanotic congenital heart disease, involves 4 anatomic abnormalities: right ventricular outflow tract obstruction, right ventricular hypertrophy, ventricular septal defect, and overriding aorta. Surgical repair, typically performed between 4 and 6 months of age, aims to restore normal anatomy, with valve-sparing techniques preferred when feasible.^1^ Long-term complications, including pulmonary regurgitation, right ventricular dysfunction, and arrhythmias, often arise, especially after transannular patch (TAP) repairs, which frequently lead to chronic pulmonary regurgitation and right ventricular dilation.^2^ This case illustrates a unique instance of pulmonary valve–sparing repair in a child who initially underwent TAP repair.

We describe a 10-year-old boy with a history of TOF, diagnosed by echocardiography on day 2 of life. The patient underwent TAP repair at 6 months. Postoperative course was uneventful, with echocardiography revealing no right ventricular outflow tract obstruction or residual intracardiac shunts, but severe pulmonary regurgitation developed over time, accompanied by right ventricular dilation. Eight years after surgery, magnetic resonance imaging showed severe pulmonary regurgitation with a regurgitant fraction of 48% to 50%, right ventricular end-diastolic volume index of 171 mL/m^2^, and right ventricular ejection fraction of 47%. The patient remained asymptomatic but met the criteria for surgical intervention on the basis of magnetic resonance imaging and electrocardiography findings (QRS duration of 170 ms).

Preoperative imaging revealed intact native pulmonary valve leaflets, leading to a valve-sparing repair strategy (Figure; Video 1). During surgery, all 3 leaflets were preserved, and the TAP was resected, allowing the reconstruction of the native main pulmonary artery. Postoperative echocardiography in the parasternal long-axis (Video 2), subcostal transverse (Video 3), and parasternal short-axis views (Video 4) demonstrated well-coapting pulmonary valve leaflets with mild regurgitation, and the patient had an uneventful recovery. At the 2-week follow-up, echocardiography indicated a decrease in right ventricular size, trivial pulmonary regurgitation, and right bundle branch block with reduced QRS duration.

**Figure:**
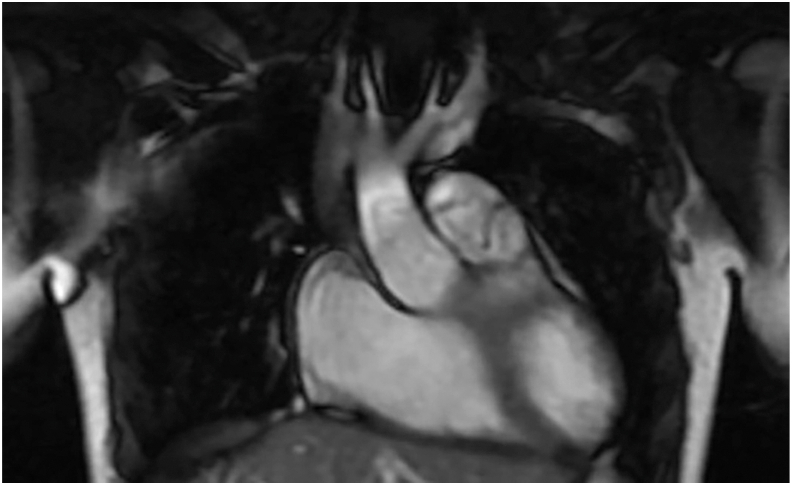
Preoperative cardiac magnetic resonance image, coronal view, showing discrete pulmonary valve leaflets.

## Comment

Whereas valve replacement remains the standard for managing severe pulmonary regurgitation, valve-sparing techniques offer several benefits, particularly in pediatric patients. By preserving native valve tissue, valve-sparing repair allows better long-term outcomes, including reduced reintervention rates and avoidance of the complications associated with prosthetic valves, such as the need for anticoagulation.^3^

A small body of literature has reported cases of pulmonary valve repair after TAP repair. Mainwaring and coworkers^4^ described 5 patients who underwent TAP repair in infancy and later required pulmonary valve repair. Unlike our case, all patients in their series had bicuspid valves, and their right ventricular end-diastolic volume index ranged from 130 to 165 mL/m^2^ compared with our patient’s 171 mL/m^2^. Another study by Papadopoulos and colleagues^5^ reported secondary pulmonary valve repair in 7 patients, employing various surgical techniques, including leaflet extension with use of autologous pericardium. In our case, all 3 native leaflets were preserved without requiring additional prosthetic material.

Park and coworkers^6^ demonstrated success in using polytetrafluoroethylene or autologous pericardium to extend native pulmonary leaflets in patients with insufficient leaflet tissue. In contrast, our patient had intact tricuspid valve leaflets that were preserved during the repair.

Pulmonary regurgitation after TAP repair remains common, with studies reporting up to 40% of patients requiring valve replacement during long-term follow-up.^7^ Chronic pulmonary regurgitation is associated with decreased survival, right ventricular dysfunction, and arrhythmias in the second decade after TOF repair.^8^ Pulmonary valve–sparing strategies, as employed in our case, provide an alternative to valve replacement and have demonstrated potential for improved long-term outcomes, although more data are needed to confirm their durability.

Another point to consider is at the time of the initial repair. It is common to resect existing pulmonary valve leaflets, particularly ones that are thickened or dysplastic, to ensure a wide-open right ventricular outflow tract. However, a transannular incision that preserves the native pulmonary valve leaflets, dividing the annulus at the anterior commissure and leaving the existing leaflets in situ, allows future pulmonary valve repair, as was done in this case. If feasible, leaving native pulmonary valve leaflets intact at the initial repair preserves surgical options for the future.

This case highlights the importance of careful preoperative imaging in planning for pulmonary valve repair after TOF repair. Preserving the patient’s native pulmonary valve leaflets allowed a durable repair and avoided the complications associated with valve replacement. Valve-sparing techniques should be considered more frequently, particularly in pediatric patients, to reduce the risk of reintervention and to improve long-term outcomes. Further studies are needed to compare the long-term efficacy of valve-sparing repair vs replacement, but this case contributes to the growing body of evidence supporting valve-sparing interventions in patients with TOF.
